# Antibiotic Treatment, Mechanisms for Failure, and Adjunctive Therapies for Infections by Group A *Streptococcus*

**DOI:** 10.3389/fmicb.2021.760255

**Published:** 2021-11-04

**Authors:** Anders F. Johnson, Christopher N. LaRock

**Affiliations:** ^1^Microbiology and Molecular Genetics Program, Graduate Division of Biological and Biomedical Sciences, Laney Graduate School, Emory University, Atlanta, GA, United States; ^2^Department of Microbiology and Immunology, Emory University School of Medicine, Atlanta, GA, United States; ^3^Division of Infectious Diseases, Department of Medicine, Emory University School of Medicine, Atlanta, GA, United States; ^4^Emory Antibiotic Resistance Center, Atlanta, GA, United States

**Keywords:** group A *Streptococcus*, *Streptococcus pyogenes*, antibiotic resistance, treatment failure, experimental therapeutics

## Abstract

Group A *Streptococcus* (GAS; *Streptococcus pyogenes*) is a nearly ubiquitous human pathogen responsible for a significant global disease burden. No vaccine exists, so antibiotics are essential for effective treatment. Despite a lower incidence of antimicrobial resistance than many pathogens, GAS is still a top 10 cause of death due to infections worldwide. The morbidity and mortality are primarily a consequence of the immune sequelae and invasive infections that are difficult to treat with antibiotics. GAS has remained susceptible to penicillin and other β-lactams, despite their widespread use for 80 years. However, the failure of treatment for invasive infections with penicillin has been consistently reported since the introduction of antibiotics, and strains with reduced susceptibility to β-lactams have emerged. Furthermore, isolates responsible for outbreaks of severe infections are increasingly resistant to other antibiotics of choice, such as clindamycin and macrolides. This review focuses on the challenges in the treatment of GAS infection, the mechanisms that contribute to antibiotic failure, and adjunctive therapeutics. Further understanding of these processes will be necessary for improving the treatment of high-risk GAS infections and surveillance for non-susceptible or resistant isolates. These insights will also help guide treatments against other leading pathogens for which conventional antibiotic strategies are increasingly failing.

## Introduction

*Streptococcus pyogenes* (group A *Streptococcus*, GAS) is a ubiquitous human pathogen responsible for over half a million deaths per year worldwide (Carapetis et al., 2005). No vaccine exists, and current treatment depends on conventional antibiotics and symptom management. While the β-lactam penicillin remains the antibiotic of choice for mild to moderate infections, severe or prolonged infections require additional measures for effective clearance. The standard recommendation is to utilize the lincosamide clindamycin in combination with penicillin (Stevens et al., 2014). Any resistance is a serious issue because of the reliance on these antibiotics, so surveillance is important. GAS has no resistance to penicillin, but treatment failure remains a major concern. Clindamycin has been very effective, but the global rates of resistance continue to rise and make the implementation of universal guidelines a challenge. Emergent challenges and opportunities for the treatment of GAS are the focus of this review.

## Group A *Streptococcus* Infections

GAS colonizes the nasopharynx, where it can cause disease, disseminate to other sites in the body, and transmit to other humans. GAS is isolated from this site in 12–24% of healthy children and in 37% of those with a sore throat (Shaikh et al., 2010). Pharyngitis, or strep throat, is the most common disease caused by GAS and is estimated to occur more than 600 million times per year (Carapetis et al., 2005). The common symptoms of pharyngitis are a sore throat, fever, enlarged tonsils, and coughing with throat pain, induced by pro-inflammatory exotoxins secreted by GAS (Dan et al., 2019; LaRock et al., 2020). Some individuals are susceptible to recurring pharyngitis (Dan et al., 2019), which may be prevented with tonsillectomy, although 33% of children lacking tonsils are still colonized by GAS (Roberts et al., 2012). GAS exotoxins also promote colonization of the skin and more serious invasive infections and are major drivers of pathogenesis (Wilde et al., 2021a).

Antibiotics remain necessary since fatal complications may occur from untreated infection. Famously, untreated pharyngitis can lead to scarlet fever, an inflammatory disease with resurging outbreaks (Davies et al., 2015; Park et al., 2017; Lynskey et al., 2019), and fatality rates up to 30% (Quinn, 1989). Scarlet fever is mediated by the streptococcal pyrogenic exotoxin superantigens, which induce an inflammatory cytokine storm (Shannon et al., 2019). In the bloodstream, superantigens are responsible for streptococcal toxic shock syndrome (STSS), a multi-organ disease with a fatality rate up to 44% (Lamagni et al., 2008; Wilkins et al., 2017). STSS often co-occurs with necrotizing fasciitis, an invasive infection of the skin (Low, 2013) where surgery within 24 h is often necessary for survival due to tissue damage and bacteremia (Olsen and Musser, 2010). Untreated GAS infections further have the risk of immune sequelae such as rheumatic fever, where the immune system mistakenly recognizes host tissue as foreign antigens (Cunningham, 2000; Hurst et al., 2018). When targeted toward the heart, this results in rheumatic heart disease, a chronic condition that is a major cause of GAS morbidity and mortality (Walker et al., 2014). The risk of any of these complications is thus limited when GAS infections are rapidly treated.

**FIGURE 1:**
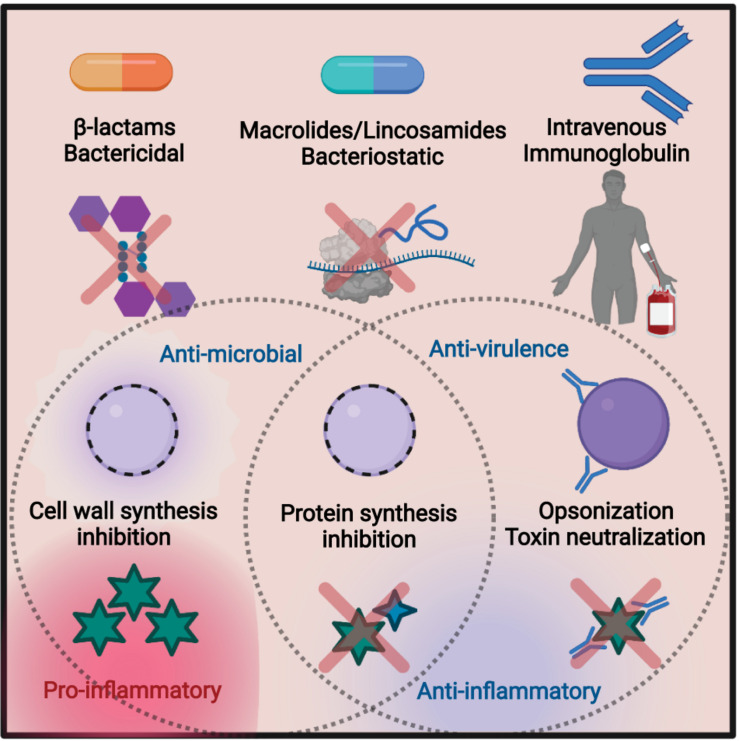
Summary of the treatment methods discussed in this review. Bactericidal β-lactams such as penicillin target the peptidoglycan of the cell wall, leading to cell lysis. This can lead to an efflux of virulence factors and other cellular proteins, resulting in inflammation. Macrolides and lincosamides are bacteriostatic, blocking protein synthesis by targeting the bacterial ribosome. Preventing toxin synthesis works to reduce inflammation. Intravenous immunoglobulin (IVIG) is an infusion of pooled antibodies from human donors, which works to induce opsonization and neutralize toxins, reducing inflammation. Figure made in biorender.

The β-lactam penicillin remains the gold standard of antibiotic treatment for many GAS infections (Stevens et al., 2014). β-lactams target penicillin-binding proteins (PBPs) to block peptidoglycan cross-linking in metabolically active bacteria, leading to bacterial death (Figure 1; Wilke et al., 2005). Despite extensive use for decades, there has been minimal change in the susceptibility of GAS to penicillin (Macris et al., 1998). Discovered in 1928 by Alexander Fleming, penicillin was brought to clinical trials in 1941. It did not take long for resistance to be observed. Penicillinase-producing *Escherichia coli* were observed in 1940, and strains of penicillin-resistant *Staphylococcus aureus* were clinically found in 1942, with 80% resistant by the end of the 1960s. Semi-synthetic versions of penicillin such as methicillin were in response; however, it would only take 20 years for methicillin resistance to become endemic (Lobanovska and Pilla, 2017).

## Non-Antimicrobial Antibiotic Effects

In animal models and human infection, clindamycin is also effective against severe GAS infection (Coyle, 2003; Carapetis et al., 2014). Clindamycin is a semi-synthetic lincosamide antibiotic that targets the 50S subunit of the ribosome (Spízek and Rezanka, 2004). Inhibition occurs through blocking of the peptidyl transferase reaction, preventing protein synthesis in susceptible pathogens, commonly Gram-positive cocci of *Streptococcus*, *Staphylococcus*, and *Clostridium* species (Stevens et al., 1987). Clindamycin is bacteriostatic and can limit the production of toxic proteins and virulence factors independent of its effects on growth (Figure 1; Schlievert and Kelly, 1984). This is also true for GAS (Mascini et al., 2001), where clindamycin inhibition of M protein synthesis promotes phagocytic killing (Gemmell et al., 1981) and inhibition of superantigens and other toxins (Sriskandan et al., 1997; Mascini et al., 2001) can mitigate septic shock (Schlievert and Kelly, 1984). Similar anti-toxin effects have been described for *Clostridium perfringens* (Stevens et al., 1995) and *Clostridioides difficile* (Zarandi et al., 2017).

Because of their efficacy, both penicillin and clindamycin are recommended as of 2014 by the Infectious Diseases Society of America guidelines for necrotizing GAS infections (Stevens et al., 2014). They should be used in combination with surgical interventions. Due to a mortality rate of 30% or higher from severe symptoms, treatment should be rapid to minimize risk of death (Stevens et al., 1989). While penicillin and clindamycin are not antagonistic when prescribed together, there is no inherent bactericidal benefit to using both (Stevens et al., 1998). However, the added benefits of clindamycin may come from ribosome inhibition reducing the development of toxin-mediated symptoms like STSS (Sartelli et al., 2018). Since penicillin treatment can lead to lysis and toxin release (Coyle, 2003), protein synthesis inhibitors like clindamycin (Kishi et al., 1999) that decrease toxin production can help mitigate excessive immune stimulation (Coyle, 2003). It remains to be determined whether adjunctive use of additional antibiotics improves treatment (Sunderkötter et al., 2019). For clindamycin-resistant GAS, early experimental work suggests linezolid (Oppegaard and Rath, 2020) as a suitable alternative, while gentamicin is also suggested as a potential candidate, albeit with potential toxicity (Andreoni et al., 2017).

## Antibiotic Resistance

GAS develops resistance to clindamycin by two primary mechanisms: target site modification or efflux pumps. Methylation of clindamycin target sites on the 23S RNA by ErmA, ErmC, or enzymes are most common (Seppälä et al., 1998). Isolates with this mechanism can either have constitutive or inducible resistance to clindamycin (Lewis and Jorgensen, 2005). Inducible resistance can result in treatment failure, as inducible clindamycin resistance is undetectable unless macrolides are also present (Lewis et al., 2014). Efflux pumps are a common resistance mechanism, such as *msrA* and *mefA* involved in macrolide resistance (Clancy et al., 1996). Despite the structural similarity of clindamycin and macrolides, these pumps have shown greater efficacy against macrolides (Sutcliffe et al., 1996). *Staphylococcus* species may also enzymatically inactivate clindamycin through LinA (Matsuoka, 2000). Due to the frequency of antibiotic resistance genes being plasmid mediated, there is concern of horizontal gene transfer generating new resistant strains (Ben Zakour et al., 2015).

Clindamycin resistance in the United States is on the rise, from an estimated 0.5% in 2003 (Richter et al., 2005) to currently as high as 15% in pediatric populations (DeMuri et al., 2017). Isolates from invasive infections are more commonly resistant, increasing from 2% to over 23% in this time (Fay et al., 2021). The resistance rates are geographically variable; in China, resistance may approach 95.5% (Stevens and Bryant, 2017), where over a similar period, northern Europe rates approximated 1% (Bruun et al., 2021). Despite the rapid change in resistance trends and the emergence of potentially hypervirulent, resistant strains, the recommendation remains: continue the use of protein synthesis inhibitors such as clindamycin when necessary, but to be mindful and vigilant for resistant isolates (Stevens et al., 2014).

β-Lactams and macrolides are the drugs of choice for GAS and therefore have the highest concern for the development of resistance. Along with rapid increases in erythromycin and clindamycin resistance, tetracycline resistance is widespread and levofloxacin resistance is observed (Fay et al., 2021). However, the challenges with GAS treatment are still typically antibiotic failure, not intrinsic drug resistance. No resistance to vancomycin or β-lactams has been observed.

## β-Lactam Resistance Concerns

The answer to why GAS has not developed resistance to β-lactams despite extensive use and widespread resistance in related species has remained elusive. A study in 1998 found no significant change in the minimum inhibitory concentration (MIC) over time (Macris et al., 1998), and this trend has continued (Fay et al., 2021). While there have been clinical isolates with elevated penicillin MIC values reported in India, Japan, and Mexico (Amábile-Cuevas et al., 2001; Capoor et al., 2006; Ogawa et al., 2011; Berwal et al., 2019), no mechanism has been provided. In other streptococci, resistance is primarily found in PBP mutations. One proposal is that PBPs with low affinity for β-lactams are poorly tolerated by GAS (Horn et al., 1998). Consistent with this, GAS engineered to express low-affinity PBPs had growth defects, poor growth rates, and morphological abnormalities (Gutmann et al., 1981; Gutmann and Tomasz, 1982). Additional work showed that decreases in the M protein production could lead to resistance, at the cost of being avirulent (Rosendal, 1958). Taken together, this suggests that PBPs are essential to GAS biology, and changes that would support resistance are either fatal or so detrimental that survival in a clinical setting is quite difficult. This has been partially backed up by recent work showing that three or fewer amino acid changes to PBP have occurred in 99% or more of the clinically relevant GAS strains (Hayes et al., 2020).

A community outbreak of GAS in Seattle recently led to the identification of two isolates with reduced susceptibility to β-lactams (Vannice et al., 2020). These isolates had a T553K substitution within *pbp2x* and a S79F substitution within *parC* of topoisomerase. The MIC values for ampicillin, amoxicillin, and cefotaxime were higher than those of isogenic isolates, while the MIC for penicillin was unchanged. The two isolates have no confirmed direct link despite their genomes being nearly identical (Vannice et al., 2020). In the wake of these findings, there were concerns that these mutations were already worldwide. Subsequent studies have identified additional natural mutations in *pbp2x* responsible for the reduced susceptibility (Musser et al., 2020). Isogenic isolates with *pbp2x* mutations show no change in virulence in a mouse model; however, they have a potential for increased fitness (Olsen et al., 2020). These mutations are concerning because of the similarities with *Streptococcus pneumoniae*, another pathogen responsible for childhood disease (Weiser et al., 2018). Penicillin had been the antibiotic of choice for treatment, but resistance became widespread in the 1980s mutations in *pbp2x* and *pbp2b* (Grebe and Hakenbeck, 1996). One possible source of resistance was horizontal gene transfer into *S. pneumoniae* from other native oral streptococcal species such as *Streptococcus mitis* (Dowson et al., 1989). T550 in *S. pneumoniae* corresponds to T553 in GAS, suggesting that future resistance could similarly arise (Vannice et al., 2020).

## Additional Considerations With Antibiotic Treatment

A penicillin allergy is one of the few reasons to consider another drug for most GAS infections. This allergy is estimated in 8% of patients, but an IgE-mediated allergic response will only be visible in 1 in 20 people (Macy and Ngor, 2013; Macy, 2014). Allergy is often over-reported or self-diagnosed, leading to other antibiotics being prescribed unnecessarily (Sousa-Pinto et al., 2017). Vancomycin or linezolid are common alternatives for those with severe penicillin allergies (Stevens et al., 2014). Allergic reactions to clindamycin are rare; it has therefore become common as an alternative choice in instances of allergic reactions to other antibiotics (Lammintausta et al., 2002). Since infection is recurrent for many people, repeated use of penicillin may drive allergy, select for resistance in other species of microbes present, and give rise to a series of opportunistic infections by pathogens such as *C. difficile* (Johnson et al., 1999; Brindle et al., 2017).

## Mechanisms for Treatment Failure

Thus, despite *in vitro* sensitivity to many antibiotics, including universal sensitivity to penicillin, GAS remains a major public health burden. Treatment failure was first reported not long after the introduction of penicillin (Eagle, 1952) and has remained a problem ever since in both common pharyngitis and more severe invasive infections (Markowitz et al., 1993; Gillespie, 1998; Orrling et al., 2001). Death due to treatment failure is not due exclusively to lack of access to antibiotics or medical treatment because, even in resource-rich countries, invasive infections can have a high failure rate during treatment (Orrling et al., 1994). Since death is not always from overwhelming bacteremia, but rather pathological inflammation as sepsis, a bolus of antibiotic leading to massive bacterial lysis may transiently exacerbate the disease or even lead to death (Wolf et al., 2017). Individuals treated with only penicillin have also shown greater risk of recurrent tonsillitis, suggesting an inability to clear the infection fully (Brook and Hirokawa, 1985).

**FIGURE 2:**
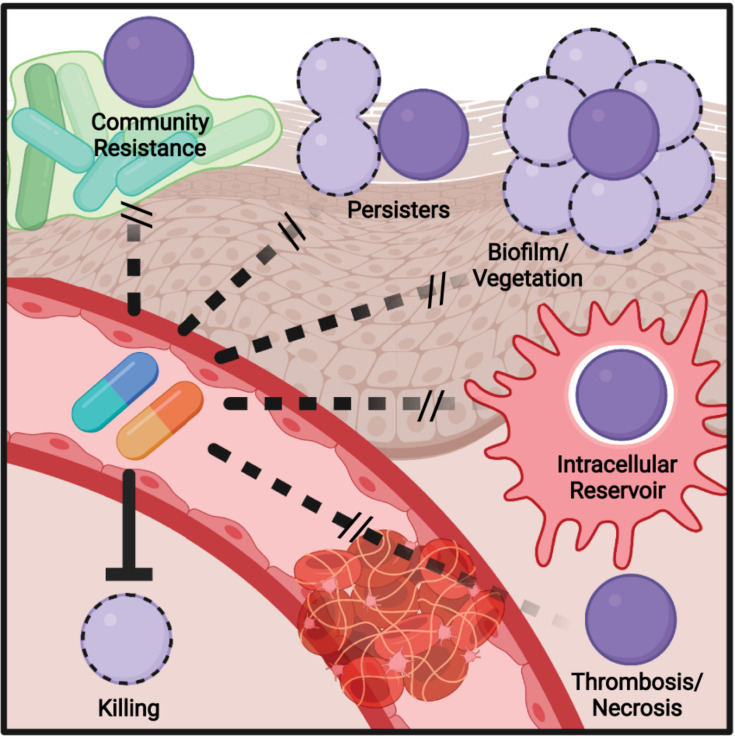
Model of mechanisms contributing to antibiotic failure during Group A *Streptococcus* (GAS) infections. Community-mediated resistance mediated by protection by endogenous microbiota is likely most prevalent during pharyngitis and not invasive infections, where GAS most often exists as a monoculture. Persisters, resistant through altered growth rates or other epigenetic states, can contribute to treatment failure of any infection. The formation of biofilms, invasion of epithelial cells, and survival within phagocytes can similarly occur during any infection and serve to shield single bacterium from antibiotic action. During invasive infections in particular, inflammation- and toxin-mediated necrosis of tissue and thrombosis of dermal vasculature can limit antibiotic perfusion, necessitating surgical removal of the infected tissue.

Bacteria can survive at antibiotic concentrations beyond a minimal bactericidal concentration (MBC) by a process known as the Eagle effect (Prasetyoputri et al., 2019). First observed in 1948 (Eagle and Musselman, 1948), it is speculated to be related to penicillin having greater efficacy on bacteria in log phase growth, as they are actively rebuilding their peptidoglycan (Eagle, 1952). During infection, resource limitation and antimicrobial immune responses slowing bacterial growth may lead to decreased antibiotic efficacy (Figure 2). This has manifested in treatment failure using the mouse model of GAS infection, where delaying penicillin treatment led to a significant reduction in survival (Stevens et al., 1988).

Community-mediated resistance (Figure 2) is another mechanism that may contribute to failure, where β-lactamases secreted by the resident microbiota in the polymicrobial environment protect sensitive pathogens, including GAS (Sorg et al., 2016; Gjonbalaj et al., 2020). One study showed that β-lactamase producers were found in 40% of pediatric patients with orofacial or respiratory tract infections (Brook, 1984), with another suggesting rates as high as 74% in the tonsils (Brook, 2009). One potential impact of clindamycin is therefore killing β-lactam-resistant species that provided protection to GAS, allowing for later reinfection (Brook and Hirokawa, 1985). The deep tissue is commonly sterile, so community resistance is more likely to play a role during pharyngitis, where there is an abundant polymicrobial community present.

Biofilms are an aggregate of bacteria encased in an extracellular matrix and contribute to the ability of many bacterial species to resist immune effectors and antibiotics. Aggregates of GAS consistent with biofilm formation have been observed in nasopharyngitis (Roberts et al., 2012) and the skin (Akiyama et al., 2003; Siemens et al., 2016). The GAS biofilm requires cell surface-anchored proteins such as pili and the serotype-specific M protein to contribute to a hydrophobic cell surface and the aggregation of GAS chains on biotic and abiotic surfaces (Frick et al., 2000; Manetti et al., 2007; Courtney et al., 2009; Matysik et al., 2020). Host proteins recruited by cell surface-anchored virulence factors further contribute to aggregation and shield GAS from antimicrobials (LaRock et al., 2015; Döhrmann et al., 2017; Alamiri et al., 2020). This protection is also extended toward antibiotics (Figure 2), with biofilm formation associated with the reduced efficacy of antibiotics *in vitro* and *in vivo* (Baldassarri et al., 2006; Marks et al., 2014; Matysik et al., 2020), including a 2,500-fold increase in penicillin tolerance in one study (Vyas et al., 2020).

While the dual role of biofilms in pathogenesis and antibiotic failure is well recognized, and a target for future therapeutics, this connection is less explored with other virulence factors. GAS can invade macrophages (Hertzén et al., 2012; Wilde et al., 2021b), epithelial (Kaplan et al., 2006), and other host cells and resist autophagy and other mechanisms to promote their intracellular growth (Barnett et al., 2013). Intracellular GAS are shielded from penicillin (Figure 2), which cannot cross the cell envelope, and the ability to invade cells is correlated with eradication failure during the treatment of pharyngitis (Sela et al., 2000). Thus, virulence factors required for cell invasion may promote penicillin failure, but not failure of cell-penetrating antibiotics such as clindamycin or erythromycin, which are more effective against intracellular GAS (Kaplan et al., 2006). The penetration of antibiotic into tissue is also a hurdle that is worsened during severe infections (Eagle, 1952; Kiang et al., 2014; Stevens and Bryant, 2017; Thabit et al., 2019). Edema, thrombosis, and tissue necrosis are pervasive during necrotizing fasciitis and other invasive GAS infections and drastically limit antibiotic perfusion (Figure 2); for this reason, surgical removal of the infected tissue is often required, even for highly antibiotic-sensitive GAS (Stevens et al., 2014). This pathology is caused directly by streptolysin O and other GAS toxins (Bryant et al., 2005).

Together, these observations suggest that the virulence factors GAS uses to escape the immune system are tied to its ability to escape antibiotics. Neutralizing antibodies and small drug inhibitors of GAS virulence factors thus have the potential to not only reduce pathogenesis and restore the effectiveness of the immune response but also to work synergistically with conventional antibiotics to break the resistance/tolerance mechanisms of GAS.

## Anti-Virulence Treatment

Since inhibiting toxin production has therapeutic benefits, neutralizing their activity may also be therapeutically useful. Intravenous immunoglobulin (IVIG) is an experimental adjunctive treatment for severe GAS infections that targets toxicity and promotes effective immune responses (Linnér et al., 2014). IVIG is generated from the pooled serum of healthy human donors and thus contains a panel of antibodies against diverse, but undefined, bacterial targets (Schwab and Nimmerjahn, 2013). These likely include major toxins and surface-anchored virulence factors (Wilde et al., 2021a). Through their neutralization (Parks et al., 2018) and increased opsonization of the bacterium, IVIG antibodies can decrease the bacterial burden and limit pro-inflammatory cytokine storms (Figure 1; Kaul et al., 1999). The repertoire of virulence factors produced by GAS is variable, as is the repertoire of specific antibodies between donors used for IVIG (Dhainaut et al., 2013), so the ability to neutralize toxins will vary between treatments and requires optimization (Norrby-Teglund et al., 1998; Schrage et al., 2006). Typical side effects include headaches or nausea (Katz et al., 2007), but there are risks of rare but severe complications (Pierce and Jain, 2003). Additional technical restrictions on using IVIG are the high cost of generation, storage requirements, and the risk of bloodborne pathogens found in any human blood.

In mice, IVIG has clear efficacy in models of STSS (Sriskandan et al., 2006) and necrotizing fasciitis (Tarnutzer et al., 2019). Because cases of severe GAS infections are rare, the opportunity to perform proper control trials is limited, and many findings may be underpowered. In some hospitals, IVIG is routinely used in tandem with clindamycin, although in one study this did not provide statistically significant improvement compared to clindamycin alone (Carapetis et al., 2014; de Prost et al., 2015). One trial was canceled due to limited enrollment, but the IVIG group had significant improvement compared to placebo (Darenberg et al., 2003), while another trial of 100 patients found no benefit over antibiotics alone (Madsen et al., 2017).

## Closing Comments

Until a vaccine is developed for GAS, antibiotics will remain essential for treating infection. The gold standard, penicillin, has been effective at treating GAS for over 80 years with no resistance, but low, consistent, rates of failure. Since other bacteria eventually gain resistance to the antibiotics commonly used for their treatment, it can be expected that GAS may eventually become resistant, which will lead to massive increases in morbidity and mortality. If the mutations in *pbpx2* of GAS continue to follow the same progression as that in *S. pneumoniae*, this may not be in the distant future (Grebe and Hakenbeck, 1996; Vannice et al., 2020). However, all mutations identified thus far are insufficient for non-susceptibility and carry a fitness cost, both of which will require additional compensatory mutations for GAS to overcome (Hanage and Shelburne, 2020). Therefore, dedicated surveillance is essential as the emergence of penicillin resistance by GAS would constitute a public health crisis.

Other methods of treatment beyond β-lactams are essential for handling severe GAS infections. While resistance is on the rise globally, clindamycin is one of the most effective treatments available alongside β-lactams to manage necrotizing fasciitis or STSS. With rapidly rising resistance, we lose this tool and will require new therapeutic strategies. As with penicillin, surveillance is crucial to determine current resistance trends. The properties that would be desired in these drugs, to complement the shortcomings of penicillin, include the targeting of vegetative bacteria in biofilms and intracellular bacteria. IVIG is a promising method to improve survival during severe infections, but it may not be a replacement for clindamycin or another effective antibiotic. Understanding how resistance develops and the global profile of resistance will ensure that new drugs can be developed and deployed in the proper locations.

## Author Contributions

Both authors listed have made a substantial, direct and intellectual contribution to the work, and approved it for publication.

## Conflict of Interest

The authors declare that the research was conducted in the absence of any commercial or financial relationships that could be construed as a potential conflict of interest.

## Publisher’s Note

All claims expressed in this article are solely those of the authors and do not necessarily represent those of their affiliated organizations, or those of the publisher, the editors and the reviewers. Any product that may be evaluated in this article, or claim that may be made by its manufacturer, is not guaranteed or endorsed by the publisher.
